# Diagnostic value of MRI and diffusion-weighted imaging in dysthyroid optic neuropathy: a cross-sectional study providing insights into predictive parameters and pathogenesis

**DOI:** 10.1007/s00234-025-03695-x

**Published:** 2025-09-17

**Authors:** Arnaud R. G. G. Potvin, Maartje M. L. de Win, Peter H. Bisschop, Michael W. T. Tanck, Robert Loontjens, Pim de Graaf, Ioana C. Lacraru, Hinke Marijke Jellema, Peerooz Saeed

**Affiliations:** 1 Orbital center Amsterdam, Department of Ophthalmology, Amsterdam UMC location University of Amsterdam, AMC, Floor D2 Room 438, Meibergdreef 9, 1105 AZ Amsterdam, The Netherlands; 2 Department of Radiology and Nuclear Medicine, Amsterdam UMC location University of Amsterdam, Meibergdreef 9, Amsterdam, The Netherlands; 3 Department of Endocrinology, Amsterdam UMC location University of Amsterdam, Meibergdreef 9, Amsterdam, The Netherlands; 4 Department of Epidemiology and Data Science, Amsterdam UMC location University of Amsterdam, Meibergdreef 9, Amsterdam, The Netherlands

**Keywords:** Dysthyroid optic neuropathy, Graves’ orbitopathy, Magnetic resonance imaging, Diffusion-weighted imaging

## Abstract

**Purpose:**

To compare magnetic resonance imaging (MRI) and clinical characteristics between patients with moderate-to-severe Graves orbitopathy (GO) and dysthyroid optic neuropathy (DON), and to assess the diagnostic value of these factors for DON.

**Methods:**

Monocentric prospective cross-sectional study. Thirty-four orbits with DON and 62 orbits with moderate-to-severe GO were included. Patients were older than 18 years, had never undergone surgical decompression, and did not receive glucocorticoid or radiotherapy within 6 months prior to participation. A standardized orbital MRI protocol was performed followed by ophthalmic and orthoptic examination within 4 weeks. Main outcome measures were: maximal radial diameter of the extraocular muscles (EOMs), signal intensity ratio of the EOMs (relative to the temporalis muscle), relative apparent diffusion coefficient (rADC) of the EOMs (relative to the temporalis muscle), apical crowding index, optic nerve stretching, clinical activity score (CAS), and duction scores. Data were analyzed using a multivariable generalized linear mixed model to identify measures associated with DON.

**Results:**

The combination of medial rectus diameter and superior rectus rADC resulted in an area under the curve of 0.937 with a sensitivity of 85.2%, specificity of 93.2%, positive predictive value of 85.2%, and negative predictive value of 93.2% at a cutoff of 0.461. Apical crowding and optic nerve stretching were not independently associated with DON.

**Conclusions:**

MRI with diffusion-weighted imaging sequences can be a useful adjunct in the diagnosis of DON. Medial rectus diameter and superior rectus rADC are independently associated with DON. Apical crowding and optic nerve stretching may be less important pathogenically.

**Key messages:**

1. What is already known? The diagnosis of dysthyroid optic neuropathy (DON) can be challenging due to the lack of validated diagnostic imaging features.
2. What this study adds:
  - Medial rectus diameter and superior rectus relative apparent diffusion coefficient measured by magnetic resonance imaging are independent predictors of DON.
  - Apical crowding and optic nerve stretching are not independently associated with DON.
3. How this study might affect research: MRI with diffusion-weighted imaging sequences can be a useful adjunct in the diagnosis of DON.

**Supplementary Information:**

The online version contains supplementary material available at 10.1007/s00234-025-03695-x.

## Introduction

Dysthyroid optic neuropathy (DON) is a serious complication of Graves’ orbitopathy (GO), with an estimated incidence of 5% to 8.6% [1–3]. While the exact mechanisms underlying DON have not been established, nerve compression and/or microvascular ischemia are probably involved [4]. Although most cases are reversible to some degree, there is a risk of permanent visual function loss. DON diagnosis is mainly based on clinical evaluation, but its diagnostic criteria remain disputed, and there is currently no gold standard. Accurate diagnosis can be challenging, especially in the early stages or in atypical cases when clinical evaluation is uninformative [5, 6].

Computed tomography (CT) is the main imaging technique used to evaluate GO in clinical practice and research. Magnetic resonance imaging (MRI) is an alternative technique that allows better detection of inflammatory activity, with better soft tissue discrimination, and can objectively quantify inflammatory activity using methods like short tau inversion recovery (STIR) and diffusion-weighted imaging (DWI) [7, 8]. These advantages are essential, as treatments are most likely to be beneficial in the active inflammatory stage [3]. Inflammation has been assessed in different orbital structures [9–12]. We hypothesized that MRI and clinical parameters could point to more soft-tissue enlargement and inflammation in patients with DON than in patients with moderate-to-severe GO, and that multivariable statistical analysis could indicate which clinical and/or imaging parameters are useful to predict the presence of DON. Additionally, we aim to characterize the diagnostic value of the parameters used.

## Methods

We performed a cross-sectional MRI study of consecutive patients treated at our multidisciplinary tertiary GO clinic from 2018 to 2021. The study was approved by the medical ethics committee of the Amsterdam UMC and followed the tenets of the Declaration of Helsinki. All patients provided written informed consent.

Only patients older than 18 years with moderate-to-severe GO or DON were eligible for inclusion. Diagnosis of GO was based on serology and clinical signs. Clinical severity was assessed using the EUGOGO clinical criteria [6]. The diagnosis of definite DON was based on the presence of GO with two or more of the following clinical criteria: reduced best-corrected visual acuity in decimal points (BCVA, ≤ 0.8 or a reduction of ≥ 0.2 compared to a previous measurement); impaired Ishihara color vision (≤ 13 of the first 17 plates correct in the 38 plate edition); visual field defect on automatic perimetry not explained by any other pathology; relative afferent pupillary defect (RAPD); and optic nerve head (ONH) swelling [13]. Exclusion criteria were previous orbital surgery, radiotherapy or glucocorticoid treatment for GO within 6 months prior to participation, unavailability of MRI, and refusal of treatment and follow-up.

In our multidisciplinary GO clinic patients underwent all examinations, including imaging, and were seen by an orbital specialist and an endocrinologist. If imaging could not be performed on the same day, it was ensured that MRI was planned within 4 weeks of the GO clinic visit. When the diagnosis of DON was equivocal, patients were re-examined shortly afterwards to assess reversibility or deterioration of clinical findings. In these cases, the clinical findings of the visit closest to the timing of MRI were used for the analyses.

### Clinical and radiological assessment

Patients were examined, treated, and followed up according to the 2016 EUGOGO guidelines for moderate-to-severe GO and DON [5], and their baseline characteristics, relevant clinical history and risk factors were recorded. Primary clinical outcomes were the clinical activity score (CAS) and ductions in degrees using a Goldmann perimeter. Secondary clinical variables included Gorman diplopia score, exophthalmometry, intraocular pressure, lid fissure, margin-to-reflex distance 1, and presence of eyelid retraction.

Subjects were scanned on a Siemens Avanto 1.5 T MRI system with a 16-channel head coil or a Philips Ingenia Elition 3 T MRI system with a 32-channel head coil. We acquired coronal T1 turbo spin echo (TSE), axial T2 TSE, coronal T1-STIR, and axial DWI series with a maximum slice thickness of 3 mm. For the DWI series we used the echoplanar imaging (EPI) RESOLVE technique with an EPI factor of 96 on the 1.5 T Siemens and the DWI TSE technique on the Philips 3 T and apparent diffusion coefficients (ADC) maps were calculated [14]. Both DWI techniques give less distortion artifacts in the orbital region compared to the standard EPI DWI technique. [15]

Two examiners (one head-and-neck radiologist with 12 years of experience and one ophthalmologist), who were blinded for the clinical information, independently scored the MRIs. The following primary parameters were assessed: diameters of the extraocular muscles (EOMs), apical crowding, optic nerve stretching, and relative ADC and STIR signal intensities of the EOMs.

Maximal radial diameters of the EOMs were measured on coronal (superior and inferior rectus muscle, and superior muscle complex consisting of the superior rectus and levator palpebrae muscles taken together) and axial (medial and lateral rectus muscle) slices. Diametric measurements of the EOMs were used as they have been proven to be equivalent to volumetric assessment and do not require sophisticated software, allowing for easy extrapolation of results to clinical practice [16].

**Fig. 1:**
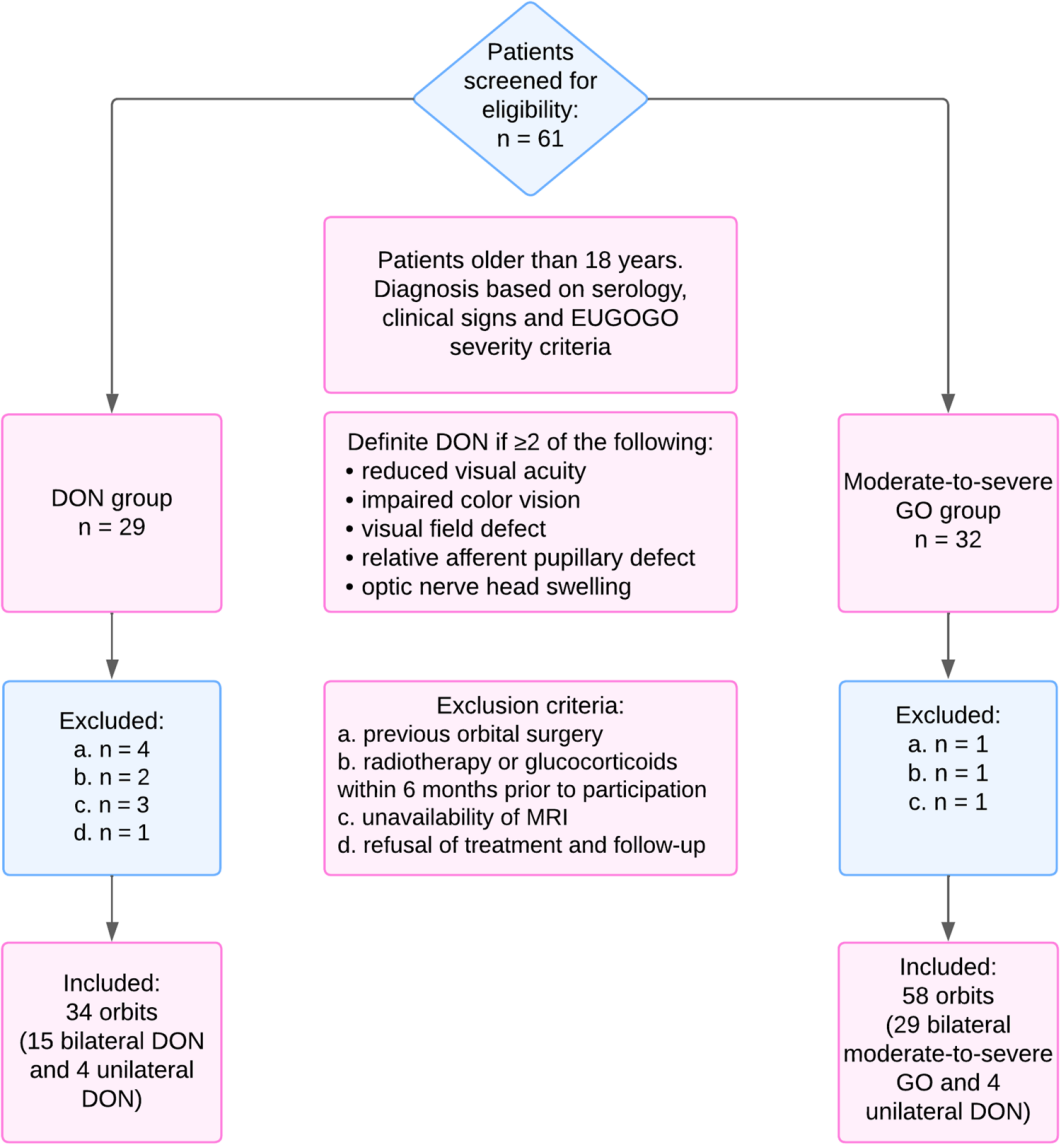
Patient selection flowchart. **Footnote:** Abbreviations: EUGOGO: European Group on Graves’ Orbitopathy; DON: dysthyroid optic neuropathy; GO: Graves’ Orbitopathy

In addition, apical crowding as described by Nugent et al. [17] was scored on the second or third coronal slice anterior to the optic canal. Optic nerve stretching (categorical classification designed by our head & neck radiologist as demonstrated in Fig. 1: Grade 0: normal curve; Grade 1: less curve; Grade 2: thinning; Grade 3 tenting of the globe) was evaluated on axial slices. For apical crowding and optic nerve stretching, disagreements between observers were resolved by consensus reading to assess these factors more reliably as pathogenic mechanisms of DON.

Finally, STIR signal intensities and ADC values of the EOMs were measured in a region of interest (ROI) within the highest signal intensity in the muscle belly, as well as the signal intensities and ADC values measured in the adjacent temporalis muscle to respectively calculate the signal intensity ratio (SIR) and relative ADC (rADC). As SIR and rADC are ratios, they allow reliable use of data from different MRI scanners and settings, and offer the capability to compare results with future studies. The temporalis muscle is not involved in GO and is structurally similar and anatomically close to the EOMs [9].

On axial T2, we measured the following secondary parameters: intracranial fat prolapse through the superior orbital fissure [18], proptosis relative to the interzygomatic line at the midbulbar level, optic nerve diameter (retrobulbar and waist portions), superior ophthalmic vein diameter, and lacrimal gland herniation relative to the interzygomatic line at the midgland level. Finally, increase of orbital fat volume and presence of edematous infiltration in the orbital fat were subjectively assessed on coronal T1 and STIR sequences respectively.

### Statistical analysis

Statistical analysis was performed using IBM SPSS Statistics for Windows, version 28.0 (IBM Corp, Armonk, NY).

Primary outcome variables were first assessed using univariable generalized linear mixed models (GLMM). Parameters significant at the *P* ≤ 0.1 level were then used to build a backward stepwise multivariable GLMM to predict the presence of DON, with statistical significance defined as *P* < 0.05. Receiver operating characteristic (ROC) analysis was performed and the area under the curve (AUC) was calculated. A cutoff value for sensitivity, specificity, positive predictive value (PPV), and negative predictive value (NPV) was determined based on the Youden index. Analyses of secondary outcomes and patient characteristics were based on univariable GLMM and odds ratios (OR) are provided when appropriate. Spearman’s correlation coefficients were calculated between CAS and the other primary outcome variables. The intraclass correlation coefficient (ICC) or kappa statistic was calculated for each of the abovementioned parameters. All *P*-values in the text, tables, and figures are two-sided, and statistical significance was defined as *P* < 0.05.

## Results

**Fig. 2:**
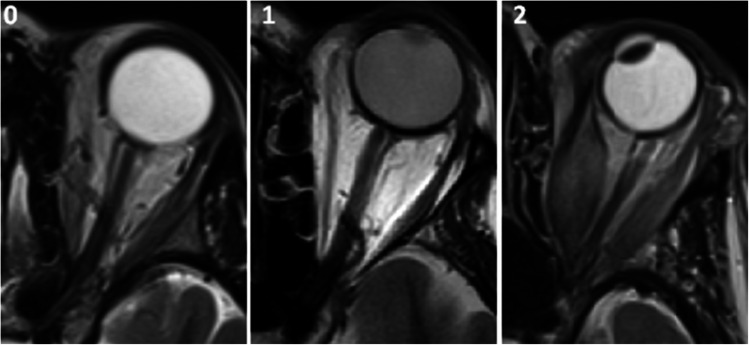
Grading of optic nerve stretching. **Footnote:** Grade 0: normal curve; grade 1: less curve or straightening; grade 2: thinning of the optic nerve. Grade 3 (tenting of the globe) was not observed in any of the patients in this study

A total of 96 orbits from 48 patients were evaluated in this study. Out of 61 patients assessed for eligibility, 13 were excluded. Figure 2 summarizes the selection process. Among the participants, 34 orbits with DON were enrolled from 15 patients with bilateral DON and 4 patients with unilateral DON, and 58 orbits with moderate-to-severe GO were included from 29 patients with bilateral moderate-to-severe GO and 4 orbits without DON from the aforementioned unilateral DON cases.

Table 1 presents baseline characteristics, relevant history and risk factors. A significantly higher proportion of DON orbits experienced deterioration of vision at onset compared with moderate-to-severe GO orbits (26.5% vs 4.8% respectively, P = 0.05. Other characteristics did not differ between groups.

**Table 1:** Baseline characteristics, history and risk factors

	**Moderate-to-severe GO**	**Dysthyroid optic neuropathy**	**P-value**
**n orbits**	62	34	
**Gender (n female (%))**	49 (79.0%)	29 (85.3%)	0.59
**Age at presentation (years)**	57.2 (14.2)	60.1 ± 11.2	0.14
**History of autoimmune disease**	8 (12.9%)	2 (5.9%)	0.44
**History of diabetes**	8 (12.9%)	8 (23.5%)	0.34
**History of smoking**			
No	19 (30.6%)	17 (50.%)	
Former	28 (45.2%)	8 (23.5%)	0.12
Current	9 (14.5%)	9 (26.5%)	0.89
Unknown	6 (9.7%)	0 (0%)	
**Thyroid status at presentation**			
Euthyroid	46 (74.2%)	20 (58.8%)	
Hyperthyroid	3 (4.8%)	3 (8.8%)	0.49
Hypothyroid	11 (17.7%)	9 (26.5%)	0.39
Unknown	2 (3.2%)	2 (5.9%)	
**Treated with radioactive iodine**	8 (12.9%)	4 (11.8%)	0.91
**Duration of eye symptoms (months)**	9 (14.5)	6 (8)	0.14
**Initial symptoms**			
Spontaneous retrobulbar pain or pressure	6 (9.7%)	10 (29.4%)	0.09
Pain or pressure with eye movement	1 (1.6%)	7 (20.6%)	0.07
Sicca (burning/stinging)	28 (45.2%)	16 (47.1%)	0.90
Eyelid swelling	13 (21.%)	11 (32.4%)	0.38
Eyelid redness	4 (6.5%)	4 (11.8%)	0.52
Proptosis	15 (24.2%)	9 (26.5%)	0.86
Diplopia	27 (43.5%)	17 (50.%)	0.66
Deterioration of vision	3 (4.8%)	9 (26.5%)	0.05
Unknown	2 (3.2%)	2 (5.9%)	0.66
**Thyroid lab**			
FT4 (pmol/l)	18.0 (5.23)	17.3 (3.97)	0.69
TSH (mU/l)	1.6 (3.33)	0.59 (7.92)	0.27
TSH-R Ab (U/l)	4.5 (23.2)	16.3 (17.5)	0.54

Baseline patient characteristics summarized as mean ± SD, median (IQR) or n (%). Statistical tests performed were univariable generalized linear mixed models.

### Univariable analysis

Table 2 lists the outcomes of univariable GLMMs of primary variables with odds ratios for the presence of DON. CAS was higher, and a higher proportion of patients had active disease in the DON group. Ductions were more restricted in the DON group. Depression and adduction, as well as CAS (both ordinal and dichotomized) were the clinical factors associated with DON in univariable analyses that reached the threshold for inclusion in the multivariable GLMM. Of the radiologic parameters, EOMs were thicker in DON orbits, and rADC values were higher. However, SIR values were higher in the moderate-to-severe group. DON orbits had a higher apical crowding index. All EOM diameters, medial rectus (MR) SIR and rADC, superior rectus (SR) rADC, inferior rectus (IR) SIR and rADC, and apical crowding were used in the multivariable GLMM.

**Table 2:** Results of the univariable generalized linear mixed models for DON

	**Moderate-to-severe GO**	**Dysthyroid optic neuropathy**	**Odds ratio (95% confidence interval)**	**P-value**
**Clinical parameters**				
CAS (score out of 7)	3 (3)	4 (2)	1.52 (1.04—2.23)	**0.03**
CAS ≥ 3	42 (67.7%)	32 (94.1%)	7.62 (0.93—62.68)	**0.06**
Ductions (degrees)				
Mean	35.82 ± 6.98	31.73 ± 5.96	0.99 (0.93—1.04)	0.66
Elevation	56 (6)	46.17 ± 8.45	1.02 (0.99—1.06)	0.18
Depression	19 (19)	17.90 ± 8.43	0.89 (0.83—0.94)	** < 0.001**
Adduction	35 ± 13	29.68 ± 9.46	0.93 (0.87—1.00)	**0.04**
Abduction	38.29 (5.17)	33.68 ± 5.86	1.01 (0.97—1.05)	0.62
**Radiologic parameters**				
Diameter (mm)				
Medial rectus	5.45 (2.13)	8.27 ± 1.71	1.74 (1.39—2.18)	** < 0.001**
Superior rectus	3.37 (1.73)	5.13 ± 1.66	1.57 (1.13—2.17)	**0.01**
Superior muscle complex	5.33 (1.71)	7.74 ± 2.19	1.65 (1.23—2.23)	**0.001**
Lateral rectus	4.72 ± 1.13	5.78 ± 1.58	1.27 (0.97—1.67)	**0.09**
Inferior rectus	7.23 (2.10)	8.78 ± 1.52	1.20 (1.01—1.41)	**0.04**
SIR*				
Medial rectus SIR	2.13 (0.62)	2.07 (1.04)	1.11 (1.01—1.22)	**0.04**
Superior rectus SIR	2.12 ± 0.48	2.07 ± 0.48	0.91 (0.81—1.02)	0.11
Lateral rectus SIR	2.04 ± 0.37	1.88 (0.64)	0.95 (0.84—1.07)	0.39
Inferior rectus SIR	2.16 (0.69)	2.10 (0.85)	1.06 (0.99—1.14)	**0.09**
rADC*				
Medial rectus	1.21 ± 0.19	1.38 (0.51)	1.17 (0.84—1.64)	**0.09**
Superior rectus	1.19 ± 0.19	1.40 (0.37)	1.24 (1.00—1.52)	**0.05**
Lateral rectus	1.13 ± 0.19	1.22 (0.46)	1.04 (0.93—1.17)	0.49
Inferior rectus	1.08 (0.20)	1.28 (0.35)	1.15 (0.98—1.34)	**0.09**
Apical crowding				
0	32 (51.6%)	6 (17.6%)		
1	27 (43.5%)	18 (52.9%)	2.31 (0.86—6.20)	**0.10**
2	3 (4.8%)	7 (20.6%)	4.67 (1.12—19.43)	**0.04**
3	0 (0.0%)	1 (2.9%)	7.79 (0.43—140.85)	0.17
Stretching				
0	7 (11.3%)	5 (14.7%)		
1	37 (59.7%)	12 (35.3%)	0.47 (0.19—1.18)	0.11
2	18 (29.%)	17 (50.0%)	1.55 (0.47—5.13)	0.47

Results summarized as mean ± SD, median (IQR) or n (%). *Odds ratio and confidence interval are reported for a unit Change of 0.1. P-values in **bold** indicate parameters included in the multivariable analysis

Of all secondary outcomes, only exophthalmometry, vertical lid fissure, and radiological proptosis (mm) were significantly different between groups (OR 1.183, *P* = 0.021; OR 0.575, *P* = 0.006; OR 1.246, *P* = 0.009, respectively). Results are shown in Online Resource 1.

### Interobserver agreement

Interobserver agreement was good (ICC 0.75–0.90; kappa 0.61–0.80) to excellent (ICC > 0.90; kappa > 0.81) for all primary outcomes, as well as for proptosis measurement and subjective EOM enlargement evaluation. Retrobulbar optic nerve diameter and superior ophthalmic vein diameter showed moderate agreement (ICC 0.50–0.75; kappa 0.41–0.60). Waist optic nerve diameter, intracranial fat prolapse, fat volume increase, and fat edema showed fair to poor interobserver reliability (ICC < 0.50; kappa 0.21–0.40).

### Multivariable analysis

**Fig. 3:**
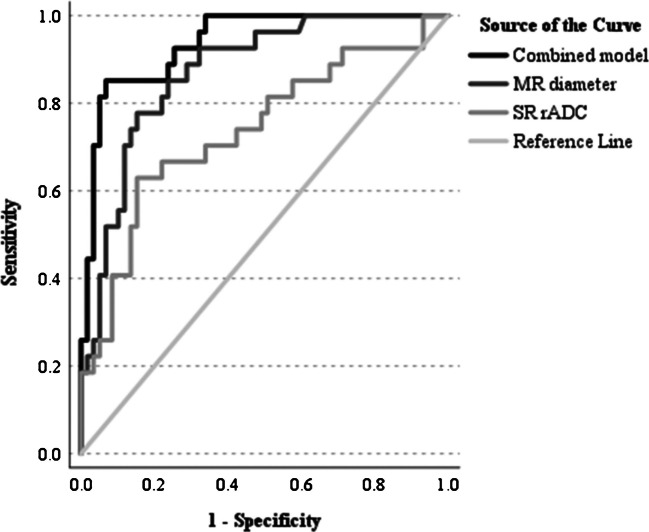
Receiver operating characteristics curve of the multivariable generalized linear mixed model for the diagnosis of dysthyroid optic neuropathy. **Footnote:** Abbreviations: MR: medial rectus muscle; rADC: relative apparent diffusion coefficient; SR: superior rectus muscle

Of all parameters included in the multivariable GLMM, only MR diameter and SR rADC were independent significant predictors of DON. This combined model (ln odds (DON = 1) = −15.437 + 0.867 × MR diameter + 6.241 × SR rADC) resulted in an AUC of 0.937 (95% CI 0.887–0.988) with a sensitivity of 85.2%, specificity of 93.2%, positive predictive value (PPV) of 85.2%, and negative predictive value (NPV) of 93.2% at a cutoff of 0.461. As a univariable predictor, MR diameter yielded an AUC of 0.857 with a sensitivity of 94.1%, specificity of 67.7%, PPV of 61.5%, and NPV of 95.5% at a cutoff of 6.01 mm. For SR rADC, a cutoff of 1.34 generated an AUC of 0.737, sensitivity of 63.0%, specificity of 84.7%, PPV of 65.4% and NPV of 83.3%. The three ROC curves are illustrated in Fig. 3.

## Discussion

The present study was performed to compare MRI and clinical characteristics between patients with DON and moderate-severe GO and to assess their value for the diagnosis of DON. In a multivariable analysis, we found that MR diameter and SR rADC could independently diagnose DON with high accuracy.

We compared MRI-specific parameters that mirror inflammatory activity between moderate-to-severe GO and DON patients, and included the rADC in logistic regression models. The superior rectus rADC added value to the multivariable model which showed high diagnostic accuracy. Two studies have already shown a significant difference in EOM ADC values between GO patients and healthy controls, even for EOMs that seemed uninvolved on conventional MRI. There were no significant differences between active and inactive GO patients and between EOM groups with different muscle dysfunction scores [19, 20]. Here, we demonstrated the ability of this inflammatory marker to differentiate between moderate-to-severe GO and DON. In addition to depicting clinically silent inflammation possibly requiring treatment and monitoring treatment response, our results suggest that the rADC is a useful adjunct for differentiating moderate-to-severe GO from DON, which can be clinically challenging.

There is some controversy regarding whether the MR or SR is pivotal in the development of DON. It is clear that muscle volume enlargement is an important characteristic of worsening disease. A recent study described a deep-learning model that succeeded in differentiating patients with mild, moderate and severe GO using orbital volume segmentation, demonstrating progressive increases in muscle volumes with worsening disease severity [21]. Of all the EOMs, the MR was the only muscle whose diameter was independently related to DON in our study. This muscle lies most closely to the optic nerve at the orbital apex, and this proximity likely causes the MR to affect the optic nerve to a greater extent than the other EOMs, which may explain why the other EOM diameters did not independently predict the presence of DON. This was consistent with previous reports identifying the MR diameter as a significant predictor in uni- and multivariable regression models [16, 22, 23]. In contrast, some of our findings suggest a role for the superior muscle complex (SMC). Restrictive limitation of depression resulting from SR swelling was associated with DON in the univariable, but not the multivariable model. In addition, vertical lid fissure was increased in the DON group and was also related to the SMC. Some studies have shown that only the SMC cross-sectional area is an independent significant predictor of worsening visual field defects [13], and that it is significantly larger relative to the total apical soft tissue volume in patients with DON [24]. In addition, two studies determined that visual field defects in DON are mostly located inferiorly, which reflects optic nerve damage superiorly [25, 26]. In contrast, Weis et al. [22] reported ptosis as a significant predictor of DON in a multivariable model, and a smaller interpalpebral fissure length was a significant predictor only in a univariable model. In summary, we found that MR diameter as well as SR inflammation could aid in the diagnosis of DON. Whereas an increase in MR diameter can logically be linked to the development of DON, the pathogenic mechanism SR inflammation is less clear. It is possible that the higher SR rADC is only an indicator of worse SR involvement in more severe disease, rather than being a key feature in the pathogenesis of DON. It is possible that in the early stages of GO there is a more generalized enlargement and inflammation of EOMs but, as the disease progresses, superior muscle enlargement may be the driving factor of vision loss [13, 24, 27].

Apical crowding is generally believed to play an important role in the pathogenesis of DON. Several studies, mostly using CT imaging, suggested that grade 3 crowding is a sensitive indicator of DON [1, 17, 18, 28, 29]. Nonetheless, other studies called its diagnostic use into doubt, indicating a large degree of overlap between DON and moderate-to-severe GO patients and suggesting that apical crowding is not the only factor responsible for DON [30–32]. Consistent with these findings, we found considerable overlap; only one orbit in the DON group had grade 3 crowding, and apical crowding lost significance in the multivariable model. Grade 2 or higher crowding by itself may not be a relevant diagnostic indicator for DON, as approximately 70% of DON orbits in our study had grade 0 or 1 crowding. It is possible that MRI allows visualization of the perineural fat plane in more detail than CT, resulting in lower scoring and weakening the predictive power of apical crowding for DON. Overall, DON seems to result from increased orbital pressure at the apex, perhaps causing microvascular damage, rather than direct compression of the optic nerve, which cannot always be adequately assessed by grading apical crowding.

Stretching of the optic nerve is another proposed pathogenic mechanism of DON. We found that radiological proptosis, but not optic nerve stretching, was significantly increased in DON. Several studies showed that proptosis lacked diagnostic value [18, 22, 28, 33]. Although the optic nerve was significantly longer in DON patients, the optic nerve did not reach its elastic limit in DON, in one study. Greater length could merely reflect greater proptosis and not be a causative factor in DON, although elongation may add insult to injury, such as microvascular ischemia [34]. Our results add to the premise that optic nerve stretching is not a critical pathogenic mechanism of DON.

This study had the limitation of a relatively small sample size and monocentric design in common with previous studies of DON, as it remains an uncommon complication of GO. It was not possible to calculate intraobserver agreement, as all measurements were performed only once for each patient by each observer. While two different scanners were used, this should not have affected diameter, crowding and stretching measurements, as these are not measurements likely to be influenced by scanner settings. However, signal intensities (SI) and ADC values can differ between scanners. To mitigate this, we used relative SI (SIR) and ADC (rADC) compared to the temporal muscles, since ratios are more consistent across equipment, reflecting the reality of current research and clinical practice [35]. Although MRI may offer advantages over CT in certain clinical contexts, particularly in cases of diagnostic uncertainty or when assessing inflammation using advanced techniques, this study was not designed to prove superiority of MRI over CT. Thus, conclusions cannot be drawn about the relative efficacy of both modalities. Nonetheless, it is important to note that certain MRI parameters have been found to have diagnostic value for DON in other studies, in particular imaging parameters of the optic nerve. Mean T2 of the optic nerve was found to be significantly higher in DON orbits and, together with a modified muscle index, had a high diagnostic sensitivity [36]. Wu et al. reported that DON orbits had a higher water fraction of the optic nerve than GO patients, possibly reflecting oedema secondary to compression or inflammation [37]. In a follow-up study, they found that optic nerve subarachnoid space and cerebrospinal fluid volume in the optic nerve are independent risk factors of DON, and aided in the diagnosis in a multivariable regression model [38]. Finally, diffusivity and anisotropy measurements of the optic nerve differed significantly between DON and GO orbits in a study using diffusion tensor imaging [39]. It should be noted that these are advanced, time-consuming techniques that are not readily available in every center. Finally, while CT is often preferred for orbital bone visualization, MRI can provide sufficient anatomical detail for surgical planning. It should be noted, however, that MR diameter, which seems to be the most important diagnostic factor from our study, can readily be assessed with CT. Prospective studies directly comparing both techniques for the diagnosis of DON should be performed.

In conclusion, we developed a multivariable prediction model based on the MR diameter and SR rADC that can accurately diagnose DON. Assessing the MR diameter can also be helpful for clinicians relying on CT imaging. Nonetheless, DON remains a mainly clinical diagnosis. Our results also indicated that apical crowding and optic nerve stretching may be less important factors in the pathogenesis of DON than previously thought. Further research is necessary to confirm our findings.

## Supplementary Information

Below is the link to the electronic supplementary material.

Supplementary file1 (PDF 28 KB)
