# Supplementary material for: Diagnostic value of MRI and diffusion-weighted imaging in dysthyroid optic neuropathy: a cross-sectional study providing insights into predictive parameters and pathogenesis
Source: Neuroradiology. 2025 Sep 17;67(9):2563–72. doi: 10.1007/s00234-025-03695-x (PMC12546493; doi:10.1007/s00234-025-03695-x)
Supplement: Supplementary file 1 — Supplementary file1 (PDF 28 KB) [file 234_2025_3695_MOESM1_ESM.pdf]

Title: Diagnostic efficacy of Magnetic Resonance Imaging in Dysthyroid Optic Neuropathy

Authors: Arnaud RGG Potvin[1], M.D.; Maartje ML de Win[2], M.D., PhD; Peter H Bisschop[3], M.D., PhD; Michael WT Tanck[4], PhD; Robert Loontjens[1], M.D.; Pim de Graaf[2], M.D, PhD; Ioana C Lacraru[1], M.D., PhD; Hinke Marijke Jellema[1], PhD; Peerooz Saeed[1], M.D., PhD
[1] Orbital center Amsterdam, Department of Ophthalmology, Amsterdam UMC location University of Amsterdam, Meibergdreef 9, Amsterdam, The Netherlands
[2] Department of Radiology and Nuclear Medicine, Amsterdam UMC location University of Amsterdam, Meibergdreef 9, Amsterdam, The Netherlands
[3] Department of Endocrinology, Amsterdam UMC location University of Amsterdam, Meibergdreef 9, Amsterdam, The Netherlands
[4] Department of Epidemiology and Data Science, Amsterdam UMC location University of Amsterdam, Meibergdreef 9, Amsterdam, The Netherlands

Corresponding author:
Arnaud Potvin
Tel: +32 476 71 53 58
E-mail: potvin.arnaud@gmail.com

Supplementary table 1: results of the univariable generalized linear mixed models of secondary parameters for DON

	Moderate-to-severe GO	Dysthyroid optic neuropathy	Odds ratio (95% confidence interval)	P-value
**Clinical parameters**				
Exophthalmometry (mm)	21.00 (6.00)	23.63 ± 3.09	1.18 (1.03 - 1.36)	0.02
Intraocular pressure (mmHg)	16.67 ± 3.22	18.00 (6.00)	1.04 (0.97 - 1.13)	0.26
Vertical lid fissure (mm)	13.00 (3.00)	9.71 ± 1.82	0.57 (0.40 - 0.84)	0.01
MRD1 (mm)	5.64 ± 1.77	3.00 (3.00)	0.83 (0.61 - 1.14)	0.26
Gorman score				
1	13 (21.0%)	11 (32.4%)		
2	16 (25.8%)	4 (11.8%)	0.30 (0.04 - 2.05)	0.22
3	19 (30.6%)	7 (20.6%)	0.44 (0.08 - 2.35)	0.34
4	13 (21.0%)	11 (32.4%)	1.00 (0.20 - 5.05)	1.00
Scleral show upper lid	21 (33.9%)	5 (14.7%)	0.65 (0.25 - 1.72)	0.40
Scleral show lower lid	8 (12.9%)	5 (14.7%)	2.50 (0.74 - 8.48)	0.15
**Radiologic parameters**				
Proptosis (grade)				
0	9 (14.5%)	2 (5.9%)		
1	33 (53.2%)	9 (26.5%)	1.11 (0.27 - 4.62)	0.89
2	6 (9.7%)	2 (5.9%)	0.06 (0.01 - 0.39)	0.77
3	14 (22.6%)	20 (58.8%)	7.18 (1.38 - 37.23)	0.02
Proptosis (mm)	-2.42 ± 3.58	0.50 (3.65)	1.25 (1.06 - 1.46)	0.01
ON diameter (retrobulbar, mm)	5.10 (1.40)	5.75 ± 1.16	0.96 (0.70 - 1.31)	0.79
ON diametar (waist, mm)	3.10 (0.80)	3.75 (0.80)	1.26 (0.90 - 1.78)	0.18
Superior ophthalmic vein diameter (mm)	1.40 (0.45)	1.52 ± 0.40	1.87 (0.77 - 4.50)	0.17
Lacrimal gland herniation (mm)	9.10 ± 2.89	9.51 ± 3.02	1.05 (0.92 - 1.20)	0.49
Fat: volume increase	47 (75.8%)	29 (85.3%)	1.60 (0.34 - 7.60)	0.55
Fat: edematous infiltration	46 (74.2%)	32 (94.1%)	3.00 (0.76 - 11.81)	0.12
Fat: prolapse through superior orbital fissure	16 (25.8%)	12 (35.3%)	1.28 (0.52 - 3.14)	0.60

Results summarized as mean ± SD, median (IQR) or n (%). *OR and CI are reported for a unit change of 0.1.
